# A perspective on tumor radiation resistance following high-LET radiation treatment

**DOI:** 10.1007/s00432-024-05757-8

**Published:** 2024-05-02

**Authors:** Yogendra Singh Rajpurohit, Dhirendra Kumar Sharma, Mitu Lal, Ishu Soni

**Affiliations:** 1 Molecular Biology Division, Bhabha Atomic Research Centre, 2-46-S, Modular Lab, A-Block, Mumbai, 400085 India; 2 Homi Bhabha National Institute, DAE- Deemed University, Mumbai, 400094 India

**Keywords:** Linear energy transfer (LET), Photon therapy, Relative biological effectiveness (RBE), Clustered DNA lesions, Translesion synthesis (TLS)

## Abstract

High-linear energy transfer (LET) radiation is a promising alternative to conventional low-LET radiation for therapeutic gain against cancer owing to its ability to induce complex and clustered DNA lesions. However, the development of radiation resistance poses a significant barrier. The potential molecular mechanisms that could confer resistance development are translesion synthesis (TLS), replication gap suppression (RGS) mechanisms, autophagy, epithelial-mesenchymal transition (EMT) activation, release of exosomes, and epigenetic changes. This article will discuss various types of complex clustered DNA damage, their repair mechanisms, mutagenic potential, and the development of radiation resistance strategies. Furthermore, it highlights the importance of careful consideration and patient selection when employing high-LET radiotherapy in clinical settings.

## Overview

Cancer emerges as a worldwide health emergency, standing as a primary cause of both mortality and morbidity across the human population (Ferlay 2020). According to the World Health Organization (WHO), in 2020, there were approximately 18.1 million new cancer cases, resulting in 10 million deaths. The WHO predicts a staggering increase in cancer cases over the next two decades, estimating around 28 million cases by 2040. Furthermore, projections indicate 16.3 million deaths, marking a 63.7% increase from the statistics recorded in 2020 (Sung et al. 2021). Thus, these findings and estimates highlight the urgent need for effective cancer treatment strategies. A range of treatment modalities, such as surgery, radiation therapy (RT), chemotherapy, immunotherapy, targeted therapy, stem cell transplantation, natural antioxidants, nanoparticles, ablation therapy, radionics, chemodynamic therapy, ferroptosis and sonodynamic therapy, as well as multidisciplinary approaches, are utilized in the battle against cancer (Debela et al. 2021; Pucci et al. 2019; Arruebo et al. 2011; Moo et al. 2018). Among these treatments, RT is a potent method, especially when combined with other treatment modalities. In principal, RT should be effective against all types of tumor cells. However, individual cells within tumors exhibit varying degrees of sensitivity to radiation, resulting in diverse treatment outcomes (Wang et al. 2018; Baskar et al. 2012).

Biological responses triggered by radiation exposure depend on energy deposition per unit length of radiation track (keV/μm) known as linear energy transfer (LET), along with the cellular and tissue water activity and the composition of the extracellular matrix (ECM). Low-LET radiation (γ-rays and X-rays) is termed "sparsely ionizing" because it causes scattered ionizing events in various directions across space. In contrast, high-LET radiation, exemplified by protons (≤ 50 keV/µm), carbon ions (C-ions) (≤ 200 keV/µm), and alpha-emitting radionuclides (60 to 110 keV/μm), is referred to as "densely ionizing" because its ionizing effect predominantly occurs in the narrow region at the end of the primary path (Bragg peak). This results in more complex and clustered DNA damage, characterized by the presence of more than two lesions per DNA helical turn, including strand breaks, base damage, and abasic sites (Mladenova, et al. 2022; Timm et al. 2018; Kruijff et al. 2015). This feature contributes to its greater relative biological effectiveness (RBE) in causing DNA damage and cell death (Paganetti et al. 2002; Oden et al. 2017; Ray et al. 2018; Busato et al. 2022). Modern therapy techniques utilize this principle by employing spread-out Bragg peak (SOBP) (Lin et al. 2018; Akagi et al. 2023; Darafsheh et al. 2021). This approach involves employing multiple charged particle beams with partly overlapping bragg peaks, allowing for the precise delivery of effective doses to the tumor in a three-dimensional fashion. This method reduces harm to healthy tissue situated before and after the tumor, in contrast to conventional low-LET photon therapy (γ-rays and X-rays) (Ray et al. 2018).

High-LET radiation induces dense ionization events that result in a substantial formation of complex DNA damage including lethal lesions such as base alterations, single-strand breaks (SSBs), apurinic/apyrimidinic site (AP sites), double-strand breaks (DSBs), and chromosomal aberrations (Nikjoo et al. 1998; Watanabe et al. 2015; Wilkinson et al. 2023). These lesions, collectively referred to as "clustered lesions," occur in very close proximity, typically within a few nanometers (Hada and Georgakilas 2008; Sage and Shikazono 2017). Among all types of damage, clustered lesions present a significant challenge for DNA repair machinery of the cell and explain why high-LET ionizing radiation has higher RBE and highly effective, despite not necessarily causing a greater overall number of DSBs compared to low-LET radiation (Georgakilas et al. 2013; Lorat et al. 2015; Lorat et al. 2016).

Nevertheless, despite progress in radiation techniques and treatment strategies, the emergence of radiation resistance remains a significant obstacle to enhancing treatment outcomes (Busato et al. 2022; Barcellini 2022). Radiation resistance can arise from several sources, including tumor heterogeneity (differences between tumors of identical types in distinct patients, or difference among cancerous cells within a singular tumor, or difference between a primary and a secondary tumor cells), the tumor microenvironment, TLS, RGS, EMT, and epigenetic alterations within cancer cells (Dagogo-Jack and Shaw 2018). Understanding the mechanisms that drive the development of resistance is essential for surmounting this obstacle in both traditional and novel forms of high-LET RT (Sato et al. 2019). Here, we discuss radiation resistance strategies, repair approaches for clustered DNA lesions, and the possible potential for mutagenesis against clustered DNA lesions. Furthermore, we suggest that clinicians exercise the caution when deciding to use high-LET RT as a treatment for tumors instead of low-LET RT, as there may high risk of tumor resistance developing in the medium to longer term.

### Role of DNA damage repair and other molecular mechanisms in radiation resistance against low-LET radiation

Ionizing radiation (IR) exerts a direct impact on the structure of DNA by introducing DNA breaks, specifically DSBs. Additionally, it triggers secondary indirect effects such as the production of reactive oxygen species (ROS), which in turn oxidize proteins and lipids. These ROS also contribute to various forms of DNA damage, including the creation of abasic sites and SSBs (Borrego-Soto et al. 2015). Out of the diverse types of DNA damage caused by radiation therapy, DSBs are particularly harmful (Huang and Zhou 2020; Deng, et al. 2022). There are four primary DNA repair pathways: recombination repair, mismatch repair (MMR), base excision repair (BER), and nucleotide excision repair (NER). Recombination repair deals SSBs and DSBs, while mismatch repair fixes replication errors. BER addresses oxidation-induced base damage and removes AP sites, while NER corrects bulky adducts and cross-linked DNA damage.

Among these, the DSBs repair pathways, namely, nonhomologous end joining (NHEJ) involving DNA-PKs and Ku heteroduplex proteins and homologous recombination (HR) catalyzed by Rad51 and regulated by ATM and ATR kinases, are vital for mending radiation-induced DNA breaks (Dietlein et al. 2014; Dietlein and Reinhardt 2014; Visnes et al. 2018; Zhu et al. 2009). In response to DSBs caused by low-LET radiation, sensor proteins such as Rad24p (Aylon and Kupiec 2003), NBS1/hMRE11/hRAD50 complex (Zhou and Paull 2013), γH2AX (Georgoulis, et al. 2017), Ku (Abbasi et al. 2021), MDC1 (Ruff et al. 2020), and 53BP1 (Noordermeer et al. 2018), initiate downstream signaling responses mediated by sensor kinases (DNA-PKs, ATM, and ATR) (Mladenov et al. 2019a; Mladenov et al. 2019b; Li, et al. 2021; Smith et al. 2010; Goodarzi et al. 2003). Together, this leads to an initiation of the DNA damage response (DDR) triggers a cascade of complex reactions, encompassing cell cycle arrest and DNA repair processes (Marechal and Zou 2013; Cuadrado et al. 2006; Weber and Ryan 2015). In response to low-LET RT, the expression levels of different DNA damage sensors (γH2AX, 53BP1, NBS1, BRCA1/2, and Ku) have the potential to act as predictive biomarkers for assessing the outcomes of RT in cancer patients (Liu, et al. 2020; Stover et al. 2016). Replication protein A (RPA) is a crucial eukaryotic protein involved in binding to ssDNA and has roles in DNA replication, DNA damage repair, replication stress, and cell cycle regulation. RPA may contribute to radiation resistance by utilizing the ATR/Chk1 pathway to alleviate replication stress (Glanzer et al. 2014; Zou et al. 2006). Inhibition of RPA negatively affects the time bound damaged DNA repair, rendering cells radiation-sensitive. XRCC1 (X-ray repair cross-complementing 1), a key BER protein stands out as a significant predictive biomarker for radiation therapy, and mutations within the XRCC1 gene have the potential to heighten sensitivity to ionizing radiation (Hanssen-Bauer et al. 2012; Caldecott 2019; Niu et al. 2013; Eckelmann, et al. 2020). Additionally, p53 gene (a tumor suppressor gene) expression status in cancerous cells profoundly influences the efficacy of RT (Kong et al. 2021).

In addition to DDR mechanisms, poly(ADP-ribosyl)ation (PARylation) and replication stress management are key fundamental processes that play crucial roles in safeguard of genome integrity against DNA damage. PARylation is a posttranslational modification that occurs in response to DNA damage, particularly in nuclear proteins such as histones. It serves to enhance the survival of damaged proliferating cells (Kamaletdinova et al. 2019). PAR is produced from NAD + through the action of Poly (ADP-ribose) Polymerases (PARPs), a group of 18 proteins encoded by different genes (Alemasova and Lavrik 2019). These PARPs are linked to promoting tolerance to RT (Seyedin et al. 2020; Higuchi et al. 2015). After exposure to ionizing radiation, PARP-1 activates the AMPK/mTOR pathway, leading to processes such as autophagy and reduced sensitivity to radiation (Bi et al. 2018; Rose et al. 2020).

Replication stress can arise from various sources, including genome integrity perturbation by clustered DNA damage, conflicts between replication and transcription machinery, and oncogenic stress (Nickoloff et al. 2022). Certain difficult to repair clustered DNA damages activate DNA damage tolerance (DDT) pathways, where specialized polymerases like REV1, POL ζ, POL η, POL κ, and POL i bypass damage to facilitate continuous replication and radioresistance with possibilities to introduce mutations (Chatterjee and Walker 2017). Furthermore, if these underlying issues, such as DNA damage, are not promptly resolved, nucleases facilitate the restart of the fork. Several nucleases, including MUS81 (Marini et al. 2023; Chen et al. 2021), FEN1 (Zhang et al. 2022a; Vaitsiankova et al. 2022; Yang et al. 2022a), EEPD1 (Wu et al. 2015), Artemis (Betous et al. 2018), Metnase, EXO1 (Tomimatsu et al. 2017), DNA2-BLM, MRE11 (Vertemara and Tisi 2023), CtIP (Mozaffari et al. 2021), SLX1-SLX4 (Wyatt et al. 2013; Sarbajna et al. 2014), XPG (Trego et al. 2016), and XPF-ERCC1-SLX4 (Betous et al. 2018; Xu et al. 2021) are implicated in replication stress management and play roles in promoting the repair and restart of replication forks (Nickoloff et al. 2022).

Modulating the cell cycle regulation and distribution by eukaryotic cells is another crucial adaptation for the protection of genome integrity against DNA damage (Morales-Ramirez et al. 2022; Jackson 2002). Upon detecting difficult to repair DNA damage cells may either undergo apoptosis or enter a state of senescence. Conversely, when damage is reparable, cells prolong their cell cycle to facilitate DNA repair and accomplish it. DNA damage induces cell cycle arrest, particularly through the activation of G2/M checkpoints, which are orchestrated by ATM and ATR (Marechal and Zou 2013; Cuadrado et al. 2006; Barnaba and LaRocque 2021; Pai, et al. 2021). These checkpoints play pivotal roles in preventing cells from entering the mitotic phase when DNA integrity is compromised.

Moreover, resistance to low-LET radiotherapy in cancer involves various pathways, such as autophagy (Chaachouay et al. 2011; Chen et al. 2015; Chen et al. 2011; Khan et al. 2020), the NF‐κB pathway (Deorukhkar and Krishnan 2010; Li and Sethi 2010), Akt/cyclin D1/CDK4 signaling pathway (Shimura 2017; Shimura et al. 2011; Shimura et al. 2012), and the Wnt/β‐catenin pathway (Yu et al. 2021; Liu et al. 2022). Furthermore, factors contributing to this resistance encompass tumor genetics and epigenetics, tumor metabolism, the tumor microenvironment, and the presence of nonmalignant cells, including fibroblast-associated cancer cells, tumor-infiltrating lymphocytes, endothelial cells, macrophage-associated cancer cells, and cancer stem cells (Busato et al. 2022; Rycaj and Tang 2014; Peitzsch et al. 2019; Huang et al. 2023; Wu et al. 2023a) (for a more comprehensive understanding, readers are encouraged to consult the cited references). Thus, in a wider perspective, cancer cells have the capability to build resistance against traditional low-LET RT using various mechanisms.

### High-LET radiation causes difficult to repair clustered DNA damage

High-LET radiation produces clustered DNA damage resulting in complex clustered DNA lesion sites (CDS) or multiple damage sites (MDS). These sites include clustered DSBs together with base lesions or AP sites, as well as clusters of non-DSB damage comprising base lesions and ssDNA breaks (Sage and Shikazono 2017; Georgakilas et al. 2013; Goodhead and Nikjoo 1989; Sage and Harrison 2011; Eccles et al. 2011; Sai et al. 2023; Bukowska and Karwowski 2018). At the chromosomal level, chromatin compaction reduces the incidence of DNA damage caused by ionizing radiation (Tang et al. 2019). Nevertheless, repair of high-LET-induced heterochromatic region DSBs take substantial period while DSBs caused by low-LET ionizing radiation are promptly restored within 24 h, irrespective of chromatin compaction status (Lorat et al. 2015; Roobol, et al. 2020). The DNA damages caused by low-LET radiation usually dispersed throughout the genome, and repair mechanisms are highly efficient at addressing such damage (Chatterjee and Walker 2017; Tian et al. 2015). However, repair of CDS and MDS showed slower repair kinetics, resulting in inaccurate repair and chromothripsis (a solitary catastrophic incident results in the extensive reorganization of genetic material within one or few number of chromosomes), or sometimes not at all repaired (Sage and Shikazono 2017; Cortes-Ciriano et al. 2023; Voronina et al. 2020; Kozmin, et al. 2021; Danforth et al. 2022). This distinction is a pivotal reason why high-LET carbon ions exhibit a 2–threefold greater RBE than low-LET ionizing radiation (Okayasu 2012; Allen et al. 2011; Tsujii et al. 2004; Okada et al. 2010; Paganetti 2022). From this viewpoint, the heightened cytotoxicity of high-LET ionizing radiation leverages the innate shortcomings of DNA repair systems when confronted with clustered lesions. This limitation attributed to the absence of natural selective pressure that drives the development of cellular repair systems capable of efficiently repairing clustered DNA damage (Lorat et al. 2015; Asaithamby and Chen 2011). Furthermore, from a clinical perspective, the efficacy of carbon-ion radiation therapy (CIRT) relies less on oxygen compared to low-LET RT (Antonovic et al. 2014; Strigari et al. 2018). As a result, CIRT presents as an efficient therapeutic option for hypoxic tumors, found in melanoma, head and neck cancer, and pancreatic cancer, which typically demonstrate resistance to traditional radiotherapy. (Allen et al. 2011; Kamada et al. 2015; Tinganelli and Durante 2020).

Clustered complex DNA damage can be broadly divided into two primary categories: DSBs and non-DSB oxidative clustered DNA lesions (OCDLs) (Hada and Georgakilas 2008). OCDLs can be further classified into two categories: bistranded clustered lesions (where multiple lesions are closely located on both DNA strands) and tandem clustered lesions (where multiple lesions are closely positioned on the same DNA strand). Several factors influence the repair phenomenon of clustered complex damage, including the type of lesion, lesion proximity, and lesion orientation. Synthetic oligonucleotides have been employed to study OCDL repair with known DNA lesions using mammalian nuclear or whole-cell extracts and purified BER proteins (Bellon et al. 2009; Kazmierczak-Baranska, et al. 2021; Mourgues et al. 2007; Lomax et al. 2004a; Harrison et al. 1999; Eot-Houllier et al. 2005; Boguszewska, et al. 2021; Karwowski 2019, 2021). The repair efficiency of bistranded OCDLs decreases when lesions are within five bases of an AP site or SSB (Mourgues et al. 2007; Lomax et al. 2004b). Lesions such as 8-oxoG, thymine glycol (Tg), and 5,6-dihydrothymine (DHT) reduce repair efficiency and may generate DSBs by replication (Bellon et al. 2009; Lomax et al. 2004a). Repair mechanisms also differ depending on the orientation of the bistranded lesions. In cases where lesions are in the negative orientation (with the opposing strand lesion positioned 5′ to the base opposite the reference lesion), both long and short patch BER processes are engaged. Conversely, in the positive orientation (where the opposing strand lesion is positioned 3′ to the base opposite the reference lesion), short-patch BER is the dominant mechanism (Harrison et al. 1999; Eot-Houllier et al. 2005; Paap et al. 2008). The presence of nearby lesions interferes with the initial phase of the BER pathway, which involves the excision of damaged bases by DNA glycosylases. For instance, an AP site or SSB on opposite DNA strand can significantly hinder the excision of base lesions (Georgakilas et al. 2004). The rate of base lesion excision is compromised when another lesion is nearby, mainly due to interference with glycosylase binding (Harrison et al. 1999; Eot-Houllier et al. 2005; Paap et al. 2008; Singh and Das 2013). Tandem OCDLs, also exhibit compromised repair, similar to bistranded clusters (Venkhataraman et al. 2001; Jiang et al. 2009).

To deal with DSBs generated directly by high-LET radiation tracks or DSBs converted by OCDLs, error-prone processes surpass high-fidelity homologous recombination (HR) repair (Mladenova, et al. 2022). Moreover, increased DNA end resection and DNA binding characteristics of Mre11 and Ku are crucial for repair pathway decisions for complex lesions (Yajima et al. 2013; Averbeck et al. 2014; Hays et al. 2020; Wang et al. 2010). Additionally, it has been suggested that the ATM protein, partners with the Artemis repair protein, plays a pivotal role in addressing complex and "dirty / clustered" DSBs introduced by high-LET radiation (Lobrich and Jeggo 2005; Xue et al. 2009). The results also indicate that ATM reacts differently to DSBs caused by high-LET radiation than to those induced by low-LET radiation. For instance, high-LET radiation significantly reduces ATM-specific phosphorylation of pATF2 and pSMC1. In contrast, the absence of ATM results in a noticeable decrease in the phosphorylation of DNA-PKC at Thr-2609 and adversely impacts the formation of Rad51 foci (Okayasu 2012; Whalen et al. 2008). Together, these findings suggest that the presence of clustered DNA lesions, whether bistranded or tandem, or DSBs, can hinder and/or alter the repair machinery's approach to repairing them, potentially leading to an increased lifetime of these lesions or conversion to more severe DNA lesions such as DSB, which poses a difficult task for the repair machinery.

### Clustered DNA damage implicated by high-LET radiation has high mutagenic potential

The biological ramifications of OCDLs in mammalian cells are complex and influenced by various factors. The damage caused by OCDLs is primarily repaired through BER pathway (Bellon et al. 2009; Mourgues et al. 2007; Byrne et al. 2009; Cunniffe et al. 2014a), and studies have revealed that specific glycosylases, such as MutY, may determine the mutagenic outcome of clustered base damage (Bellon et al. 2009; Kazmierczak-Baranska, et al. 2021; Byrne et al. 2009; Shikazono et al. 2006). Furthermore, bigger risks arise when bistranded / tandem cluster lesions are converted to DSBs. Studies indicate that levels of repair enzymes, DNA replication status, density of non-DSB damage, and the complexities of the BER pathway may dictate whether these lesions are converted into DSBs or undergo repair without such conversion (Sage and Shikazono 2017; Nikitaki et al. 2016). A firefly luciferase reporter assay uncovered that bistranded clusters featuring opposing furans could transition into DSBs within mammalian cells, with the predominant activity observed from the class II AP endonuclease targeting these lesions. In mammalian cells, other bistranded clusters, such as uracil or 8-oxoG, are not converted to DSBs (Malyarchuk et al. 2009). Additionally, there is only scant evidence suggesting that clustered uracil lesions may be converted into DSBs in mammalian cells (Malyarchuk and Harrison 2005). Why bistranded furans clusters are converted to DSBs more frequently than uracil or 8-oxoG lesions is explained by the concept of “passing the baton”. This involves the cooperation of short BER pathway enzymes, such as DNA glycosylase, XRCC1, AP endonuclease (Ape1), DNA pol β, and DNA ligase 1, which interact to transfer repair intermediates to subsequent repair enzymes, preventing the premature release of repair intermediates. However, disruptions to this seamless transfer, or "passing the baton", can occur when dealing with damage types such as clustered furans that necessitate the involvement of long BER pathway instead short BER pathway enzymes (Wilson and Kunkel 2000).

Together, Bistranded / tandem cluster lesions remain unrepaired, they may encounter replication forks during DNA replication, potentially causing replication-induced DSBs or DNA mispairing, which can eventually lead to collapse of replication or incorporation of adaptive mutations (Sage and Shikazono 2017; Sage and Harrison 2011; Eccles et al. 2011; Kozmin, et al. 2021; Cunniffe et al. 2014a; Cunniffe et al. 2014b; Hsu et al. 2022; Naldiga et al. 2020; Shikazono and Akamatsu 2020). These attributes were experimentally validated in proton-irradiated K-rasLA1 mice (Luitel et al. 2018) and high-LET space-irradiation-exposed Bcl2 transgenic C57BL/6 mice (Xie et al. 2020), where it has been observed that a higher incidence of increased carcinoma and DNA replication stress promotes lung carcinogenesis, respectively.

### Does clustered DNA damage introduced by high-LET radiation effectively thwart the radioresistance of cancer cells?

Advancements in research on synthetic clustered DNA lesions have deepened our understanding of the biological impacts linked to these lesions within cellular or tissue environments (Lomax et al. 2004a; Shikazono et al. 2006; Malyarchuk et al. 2004; Malyarchuk et al. 2003; Budworth and Dianov 2003). The clustered DNA lesions exhibit a broad spectrum of diversity and undergo various processing mechanisms influenced by factors such as base modifications, the distance between lesions, and the presence of strand breaks. The mutation spectrum associated with non-DSB clustered lesions encompasses various genetic alterations, including deletions, base substitutions, and insertions/deletions (indels) of 1–2 base pairs, primarily focused on the lesions within these clusters (Mladenova, et al. 2022; Kozmin, et al. 2021; Malyarchuk et al. 2009, 2008). The sequential handling of lesions within a non-DSB cluster results in the formation of SSBs as intermediate repair products. These SSBs can hinder the excision or repair of other lesions within the cluster. Consequently, the repair ability of clustered DNA lesions is diminished, leading to elevated lifetime of these lesions. Notably, the detrimental effects of repair intermediates can be exacerbated during replication, potentially leading to the generation of DSBs from single-stranded DNA gaps (Malyarchuk et al. 2009; Harper et al. 2010; Cannan and Pederson 2016; Kumar et al. 2023; Datta et al. 2005; Wang et al. 2014). While most DSBs caused by low-LET radiation are swiftly repaired during the G1 phase, mainly through the canonical nonhomologous end-joining (cNHEJ) pathway. The role of NHEJ-specific DNA-PKcs in repairing high-LET radiation-induced DSBs in tumor cells has shown (Anderson et al. 2010; Liu et al. 2018). The DSBs caused by high-LET radiation, particularly complex DSBs, exhibit slower repair kinetics and extend throughout the cell cycle and a substantial portion of DSBs undergo repair during the late S-phase and G2-phase through homologous recombination by altering cell cycle checkpoint regulations (Okayasu 2012; Okayasu et al. 2006; Gerelchuluun et al. 2015; Mohammadian Gol et al. 2019). Additionally, complex DSBs repaired slowly and inaccurately through alternative (Alt-EJ) or micro-homology mediated (MMEJ and SSA) DNA end joining, often resulting in deletions ranging from a few base pairs to several hundred bases (Scully et al. 2019; Wang and Xu 2017; Sallmyr and Tomkinson 2018; Hanscom and McVey 2020; Mladenova et al. 2022).

Moreover, high-LET radiation exposure leads to an increased occurrence of ssDNA gaps owing to the clustered pattern of DNA damage. The introduction of persistent ssDNA gaps can trigger cell death pathways. Therefore, many tumor cells have developed mechanisms to avoid and escape ssDNA gaps, such as slowing fork movement (Peng et al. 2018; Cong, et al. 2021), reversing replication forks for repair (Bai, et al. 2020), blocking fork elongation, increasing the presence of RPA, and gaining access to translesion synthesis (TLS) to ensure continuous replication without gaps (Igarashi et al. 2023; Fu et al. 2024; Nayak, et al. 2020; Nayak, et al. 2021; Tonzi and Huang 2019; Saldanha, et al. 2023; Wu et al. 2023b; Li et al. 2019; He et al. 2022; Park et al. 2022; Venkadakrishnan et al. 2023). All of these replication gap suppression (RGS) mechanisms involve distinct players that facilitate ssDNA gap avoidance and may contribute to tumor resistance to high-LET radiation. Recent research has highlighted the role of TLSs in adaptive mutability to evade targeted drugs against EGFR/BRAF (Crisafulli and Siravegna 2023; Russo et al. 2019). The unrepaired complex DSBs lead to cell death, primarily because cells are unable to complete the mitotic process. However, a significant fraction of cells proceed through the cell cycle checkpoint despite the presence of unrepaired clustered DNA lesions, leading to an increased occurrence of chromatid and chromosomal aberrations including breaks in chromatids and chromosomes (Mladenova, et al. 2022; Sadeghi Moghadam et al. 2023). Notably, dynamic movement of DSB repair foci toward less densely packed chromatin, and their aggregation with other repair centers may predispose them to erroneous rejoining, particularly during the late S and G2-phases. Consequently, compared with those exposed to low-LET radiation, cells that survive exposure to high-LET radiation exhibit significant translocations, sister chromatid exchanges, and gross chromosomal abnormalities (Mladenova, et al. 2022; Timm et al. 2018). This leads to more pronounced and intricate reorganization of the chromosomes. Additionally, a study underscores the particular types of DNA damage caused by radiation that evade repair mechanisms, resulting in clustered mutations in germ cells (Adewoye et al. 2015). Thus, ionizing radiation, particularly high-LET radiation, causes additional ssDNA gaps, mutations, and complex clustered DNA damage, leading to the killing of the majority of tumor cells (Du et al. 2022). However, a small fraction of radioresistant tumor cell can survive with increased chromosomal abnormalities and adaptive clustered mutations resulting in radiation resistance against high-LET RT (Sage and Shikazono 2017; Sage and Harrison 2011; Adewoye et al. 2015; Nickoloff et al. 2020).

### High-LET radiation therapy: harnessing its potential with caution

RT stands as an invaluable asset in cancer treatment, providing benefits to approximately half of all cancer patients. The clinical applications of high-LET radiation have seen a worldwide expansion, notably in proton and hadron therapy (Mohamad, et al. 2017; Yang et al. 2018; Hayashi et al. 2019; Tomizawa et al. 2023; Shiba et al. 2022a; Shiba et al. 2023; Musha et al. 2022; Kiseleva, et al. 2022). Despite the increasing utilization of high-LET radiation in clinics, there remains a lack of comprehensive understanding regarding the biological mechanisms underlying its effects. The remarkable feature of high-LET radiation lies in its ability to generate clustered OCDLs sites, and DSBs. The present therapeutic strategy involving the use of high-LET radiation combined with inhibitor therapy has shown initial benefits. Clinical evidence supports its effectiveness in treating various nonsquamous cell histologies, including adenocarcinoma, malignant melanoma, adenoid cystic carcinoma, hepatoma, early-stage and locally advanced non-small cell lung cancer, and bone/soft tissue sarcoma. (Mohamad, et al. 2017; Yang et al. 2018; Hayashi et al. 2019; Tomizawa et al. 2023; Shiba et al. 2022a; Shiba et al. 2023; Musha et al. 2022; Kiseleva, et al. 2022). Synergistic approach of high-LET RT alongside targeted inhibitors of DNA repair pathways (such as BER, HR, and NHEJ) have been adopted (Cesaire et al. 2019; Srivastava et al. 2018; Hirai et al. 2012; Fujisawa et al. 2015; Ma et al. 2015; Bright et al. 2022). Alternatively, targeting immune checkpoint inhibitors (anti-PD-1/PD-L1 and anti-CTLA4) is explored to counteract resistance (Zhang et al. 2022b; Mondini et al. 2020; Bernal, et al. 2024). Furthermore, high-LET RT option considered effective against hypoxic tumors (Kabakov and Yakimova 2021). However, cancer cells adapted to hypoxic conditions, often due to deregulation of hypoxia-inducible factor-1 (HIF-1) (Yeo et al. 2017; Dongre and Weinberg 2019), or heat shock factor 1 (HSF1) / heat shock protein 90 (HSP90) (Dai 2018). Consequently, small-molecule inhibitors of HIF-1 (Gameiro et al. 2016; Zhang et al. 2015), HSF1 (Schilling et al. 2015), or HSP90 (Kudryavtsev et al. 2017) demonstrate a radiosensitizing effect on hypoxic tumors treated with high-LET radiation (Li et al. 2016; Lee et al. 2016). Additionally, RT combined with inhibitors targeting the PI3K/AKT/mTOR or HIF-1α pathways has been shown to enhance the radiosensitivity of endometrial cancer (Miyasaka et al. 2015; Song et al. 2024). This enhancement is achieved through the reducing autophagy, induction of apoptosis, inhibition of EMT, as well as suppression of NHEJ and HR repair mechanisms (Mardanshahi et al. 2021; Chang et al. 2014; Chang et al. 2013). Thus, combined approach may induce systemic antitumor immune responses, presenting a promising strategy for treating metastatic and difficult to treat cancer.

**Fig. 1:**
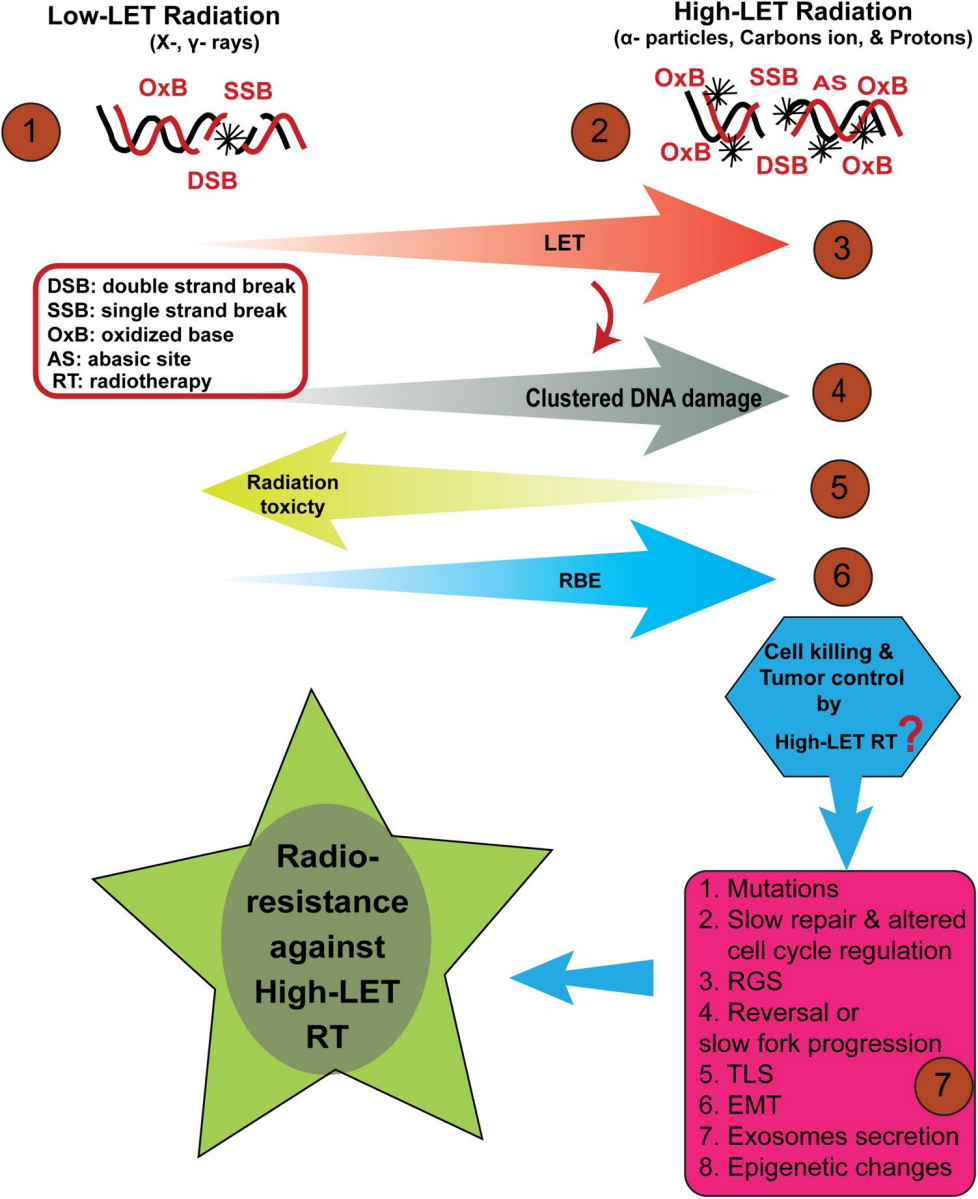
The biological effects and possible radiation resistance mechanisms against high-LET radiotherapy (RT). Cellular responses to both low and high-LET radiation offer valuable insights into the biological ramifications. Low-LET radiation (X-rays, γ-rays) causes sparsely distributed DNA damage (DSBs, SSBs, and oxidized bases) (1) in the genome, while high-LET radiation (α-particles, carbon ions, and protons) causes densely distributed clustered complex DNA damage (2). With increasing linear energy transfer (LET) (3), the density of clustered DNA damage increases markedly (4), while the reduction in radiation toxicity to healthy tissues is observed (5). However, the relative biological effectiveness (RBE) improves substantially (6). The cell killing and tumor control by high-LET RT are threatened by numerous molecular mechanisms (7). The possible mechanisms could be mutations incorporation, slow repair and altered cell cycle regulation, replication gap suppression (RGS), reversal or slow fork progression, translesion synthesis (TLS), epithelial‐mesenchymal transition (EMT) activation, release of exosomes, and epigenetic changes, which might confer radioresistance to tumor cells against high-LET RT, and may potentially, reduce the effectiveness of high-LET RT

Although high-LET RT has been shown to be beneficial over low-LET RT, apprehension and caution is warranted. Existing experimental evidence suggests that exposure to high-LET radiation is associated with an increased likelihood of cancer incidents (Luitel et al. 2018; Xie et al. 2020; Datta et al. 2013; Patel et al. 2020). Furthermore, high-LET RT has been shown to induce elevated levels of mutations, genomic instability, and chromosomal aberrations (Masumura et al. 2002; Yatagai et al. 2002), as well as elevated oxidative stress (Suman et al. 2018; Suman et al. 2013). Moreover, predictions from space exploration studies indicate a two-fold increase in excess relative risk (ERR) for male liver and female breast cancer due to high linear energy transfer (LET) radiation (Cucinotta 2022). High LET radiation also constitutes the primary source of uncertainty in galactic cosmic ray (GCR) studies (Cucinotta et al. 2020). The radioresistance observed against high-LET RT may be attributed to various damage bypass or avoidance mechanisms (Igarashi et al. 2023; Fu et al. 2024; Nayak, et al. 2020, 2021; Tonzi and Huang 2019; Saldanha, et al. 2023; Wu et al. 2023b; Li et al. 2019; He et al. 2022; Park et al. 2022; Venkadakrishnan et al. 2023; Nusawardhana et al. 2024), induction of autophagy (Chaachouay et al. 2011; Chen et al. 2015; Chen et al. 2011; Khan et al. 2020), RGS mechanisms (Cantor 2021; Cantor and Calvo 2017; Cong et al. 2024), replication fork reversal/slow movement (Zellweger et al. 2015; Bi 2015), EMT activation (Wu et al. 2023a), release of exosomes (Jokar, et al. 2022; Li et al. 2021a), and epigenetic changes (Kennedy et al. 2018; Perdyan et al. 2024; Tomsia et al. 2024) (Fig. 1). Consequently, the mutagenic potential of high-LET radiation appears to be transiently or persistently heightened. Concerns also arise regarding the delayed repair of clustered DNA damage, which could lead to mutations and the generation of DSBs from halted replication forks, particularly in rapidly dividing tumor cells. Additionally, clustered DSBs may undergo adaptive mutagenic repair, resulting in large and intricate deletions, as observed in cultured cells exposed to high-LET radiation (Michalettou, et al. 2021). Furthermore, genome loss contributes to the genomic instability of tumor cells, potentially leading to the death of the majority of tumor cells. However, rare surviving tumor cells may repopulate tumors with persistent growth due to adaptive mutability induced by high-LET radiation treatment, akin to bacterial cells. In bacterial cells, extensive generation of ssDNA gaps and unrepairable or challenging-to-repair DNA damage activate SOS responses, inducing adaptive mutagenesis (McKenzie et al. 2000; Fuchs 2016). Hence, it is crucial to carefully assess the potential of individual patient cancer cells to activate adaptive mutability and other molecular mechanisms responsible to bypass clustered DNA damage, thereby enhancing survival strategies. A comprehensive understanding of these bypass or survival strategies against clustered DNA damage is imperative on large cohorts studies over extended period for anticipating appropriate therapeutic outcomes from high-LET RT.

Furthermore, high-LET RT combined with various inhibitors approach may also pose risks to individual long-term survival due to increased genomic instability and genome restructuring. These consequences could lead to the resurgence of more aggressive tumor growth, as the tumor cells may develop adaptive resistance. Moreover, late toxicity, secondary cancers, and other adverse effects such as cataracts, fibrosis, vascular damage, immunological changes, endocrine disruptions, neurodegeneration could manifest over time (Ramaekers et al. 2011; Yang et al. 2022b; Nakashima et al. 2022; Hanna, et al. 2020; Shiba et al. 2022b; Chung et al. 2013). Additionally, while the increased energy deposition of high-LET radiation in nearby healthy tissue may not be significantly raised, the ramifications of this alteration remain undetermined (Blakely and Chang 2004). Unlike proton therapy, CIRT exhibits a fragmentation tail, wherein nuclear fragments contribute to the dose beyond the intended target area. This phenomenon introduces additional uncertainty, particularly in tissues situated distally from the target site (Malouff et al. 2020). While existing studies suggest that high-LET radiotherapy propensity to induce secondary malignancies is comparatively low compared to photon therapy, the presence of the fragmentation tail raises concerns. It is plausible that the development of secondary, radiation-induced cancers in adjacent healthy tissue may be facilitated by mutations in tumor suppressor genes (Chung et al. 2013; Dracham et al. 2018; Facoetti et al. 2019; Aherne and Murphy 2018; Kraus and Combs 2019; Yock and Caruso 2012; Mohamad et al. 2019).

Therefore, despite the increasing volume of related research and the proliferation of proton beam therapy (PBT) and/or CIRT centers, the clinical advantages of hadron-therapy treatments compared to conventional photon RT remain uncertain (Jefferson, et al. 2019). Ongoing clinical research may not resolve this uncertainty thus far due to a lack of consensus on appropriate study designs and insufficient collaboration among centers to develop comprehensive research protocols (Apisarnthanarax et al. 2018; Li et al. 2021b). Consequently, there has been a proliferation of small, inadequately designed, and poorly reported studies on this topic. These limitations may raise serious questions for the use of PBT and CIRT in experimental treatments, necessitating full disclosure of the risks and uncertainties to patients considering these therapies (Jefferson, et al. 2019; Goetz et al. 2019; Mishra et al. 2017; Fossati et al. 2018). Hence, it is imperative for clinicians and researchers to meticulously assess the therapeutic advantages of high-LET RT in a more comprehensive manner, considering the long-term suppression of tumor growth while minimizing detrimental health consequences. Researchers should focus on understanding cellular adaptive responses and strive to devise radiotherapy devices capable of inducing sparsely distributed clustered DNA damage instead of densely distributed DNA damage, thereby circumventing extreme cellular adaptive mechanisms such as RGS and the utilization of TLS for DNA damage bypass. Consequently, the notion of eradicating cancer cells without triggering their heightened adaptive mutability and facilitating DNA damage bypass should be prioritized.

## Conclusion

RT is a crucial therapeutic tool of cancer treatment, benefiting approximately half of cancer patients worldwide. High-LET radiation, notably in proton and hadron therapy, has gained global traction. However, a thorough comprehension of its biological mechanisms remains elusive. High-LET RT induces clustered DNA lesions through precision delivery of tumor radiation doses, potentially enhancing its tumor cell killing potential. Exploiting repair process vulnerabilities, such as BER and HR, alongside high-LET therapy, shows promise in augmenting tumor cell eradication. Nonetheless, caution is warranted due to cellular adaptive mutability supported by various molecular mechanisms (RGS, TLS, autophagy, EMT, release of exosomes, and epigenetic changes) which may lead to long-term toxicities and tumor relapse in the medium to longer term. Despite ongoing research, the clinical superiority of hadron therapy (high-LET) over conventional photon therapy (low-LET) remains inadequate. Addressing these uncertainties requires robust research protocols and transparent communication with patients. Therefore, a balanced evaluation of high-LET RT benefits and risks is imperative for informed clinical decisions.

## Data Availability

No datasets were generated or analysed during the current study.

## References

[CR1] Abbasi S (2021). The Ku complex: recent advances and emerging roles outside of non-homologous end-joining. Cell Mol Life Sci.

[CR2] Adewoye AB (2015). The genome-wide effects of ionizing radiation on mutation induction in the mammalian germline. Nat Commun.

[CR3] Aherne NJ, Murphy BM (2018). Radiation-induced gliomas. Crit Rev Oncog.

[CR4] Akagi T (2023). Formation of spread-out Bragg peak for helium-ion beam using microdosimetric kinetic model. Phys Med.

[CR5] Alemasova EE, Lavrik OI (2019). Poly(ADP-ribosyl)ation by PARP1: reaction mechanism and regulatory proteins. Nucleic Acids Res.

[CR6] Allen C (2011). Heavy charged particle radiobiology: using enhanced biological effectiveness and improved beam focusing to advance cancer therapy. Mutat Res.

[CR7] Anderson JA (2010). Participation of DNA-PKcs in DSB repair after exposure to high- and low-LET radiation. Radiat Res.

[CR8] Antonovic L (2014). Clinical oxygen enhancement ratio of tumors in carbon ion radiotherapy: the influence of local oxygenation changes. J Radiat Res.

[CR9] Apisarnthanarax S, Bowen SR, Combs SE (2018). Proton beam therapy and carbon ion radiotherapy for hepatocellular carcinoma. Semin Radiat Oncol.

[CR10] Arruebo M (2011). Assessment of the evolution of cancer treatment therapies. Cancers (basel).

[CR11] Asaithamby A, Chen DJ (2011). Mechanism of cluster DNA damage repair in response to high-atomic number and energy particles radiation. Mutat Res.

[CR12] Averbeck NB (2014). DNA end resection is needed for the repair of complex lesions in G1-phase human cells. Cell Cycle.

[CR13] Aylon Y, Kupiec M (2003). The checkpoint protein Rad24 of *Saccharomyces cerevisiae* is involved in processing double-strand break ends and in recombination partner choice. Mol Cell Biol.

[CR14] Bai G (2020). HLTF promotes fork reversal, limiting replication stress resistance and preventing multiple mechanisms of unrestrained DNA synthesis. Mol Cell.

[CR15] Barcellini A (2022). Ovarian cancer radiosensitivity: what have we understood so far?. Life (basel).

[CR16] Barnaba N, LaRocque JR (2021). Targeting cell cycle regulation via the G2-M checkpoint for synthetic lethality in melanoma. Cell Cycle.

[CR17] Baskar R (2012). Cancer and radiation therapy: current advances and future directions. Int J Med Sci.

[CR18] Bellon S (2009). Processing of thymine glycol in a clustered DNA damage site: mutagenic or cytotoxic. Nucleic Acids Res.

[CR19] Bernal A (2024). The current therapeutic landscape for metastatic prostate cancer. Pharmaceuticals (basel).

[CR20] Betous R (2018). DNA replication stress triggers rapid DNA replication fork breakage by Artemis and XPF. PLoS Genet.

[CR21] Bi X (2015). Mechanism of DNA damage tolerance. World J Biol Chem.

[CR22] Bi Y (2018). Radiosensitization by the PARP inhibitor olaparib in BRCA1-proficient and deficient high-grade serous ovarian carcinomas. Gynecol Oncol.

[CR23] Blakely EA, Chang PY (2004). Late effects from hadron therapy. Radiother Oncol.

[CR24] Boguszewska K (2021). How (5'S) and (5'R) 5',8-Cyclo-2'-deoxypurines affect base excision repair of clustered DNA damage in nuclear extracts of xrs5 cells? a biochemical study. Cells.

[CR25] Borrego-Soto G, Ortiz-Lopez R, Rojas-Martinez A (2015). Ionizing radiation-induced DNA injury and damage detection in patients with breast cancer. Genet Mol Biol.

[CR26] Bright SJ (2022). Targeted inhibition of DNA-PKcs, ATM, ATR, PARP, and Rad51 modulate response to X rays and protons. Radiat Res.

[CR27] Budworth H, Dianov GL (2003). Mode of inhibition of short-patch base excision repair by thymine glycol within clustered DNA lesions. J Biol Chem.

[CR28] Bukowska B, Karwowski BT (2018). The clustered DNA lesions - types, pathways of repair and relevance to human health. Curr Med Chem.

[CR29] Busato F, Khouzai BE, Mognato M (2022). Biological mechanisms to reduce radioresistance and increase the efficacy of radiotherapy: state of the art. Int J Mol Sci.

[CR30] Byrne S (2009). 5,6-Dihydrothymine impairs the base excision repair pathway of a closely opposed AP site or single-strand break. Radiat Res.

[CR31] Caldecott KW (2019). XRCC1 protein form and function. DNA Repair (amst).

[CR32] Cannan WJ, Pederson DS (2016). Mechanisms and consequences of double-strand DNA break formation in chromatin. J Cell Physiol.

[CR33] Cantor SB (2021). Revisiting the BRCA-pathway through the lens of replication gap suppression: "Gaps determine therapy response in BRCA Mutant cancer". DNA Repair (amst).

[CR34] Cantor SB, Calvo JA (2017). Fork protection and therapy resistance in hereditary breast cancer. Cold Spring Harb Symp Quant Biol.

[CR35] Cesaire M (2019). Sensitization of chondrosarcoma cells with PARP inhibitor and high-LET radiation. J Bone Oncol.

[CR36] Chaachouay H (2011). Autophagy contributes to resistance of tumor cells to ionizing radiation. Radiother Oncol.

[CR37] Chang L (2013). Acquisition of epithelial-mesenchymal transition and cancer stem cell phenotypes is associated with activation of the PI3K/Akt/mTOR pathway in prostate cancer radioresistance. Cell Death Dis.

[CR38] Chang L (2014). PI3K/Akt/mTOR pathway inhibitors enhance radiosensitivity in radioresistant prostate cancer cells through inducing apoptosis, reducing autophagy, suppressing NHEJ and HR repair pathways. Cell Death Dis.

[CR39] Chatterjee N, Walker GC (2017). Mechanisms of DNA damage, repair, and mutagenesis. Environ Mol Mutagen.

[CR40] Chen YS (2011). Autophagy inhibition contributes to radiation sensitization of esophageal squamous carcinoma cells. Dis Esophagus.

[CR41] Chen Y (2015). Combining radiation with autophagy inhibition enhances suppression of tumor growth and angiogenesis in esophageal cancer. Mol Med Rep.

[CR42] Chen S (2021). Human MUS81: a fence-sitter in cancer. Front Cell Dev Biol.

[CR43] Chung CS (2013). Incidence of second malignancies among patients treated with proton versus photon radiation. Int J Radiat Oncol Biol Phys.

[CR44] Cong K (2021). Replication gaps are a key determinant of PARP inhibitor synthetic lethality with BRCA deficiency. Mol Cell.

[CR45] Cong K (2024). FANCJ promotes PARP1 activity during DNA replication that is essential in BRCA1 deficient cells. Nat Commun.

[CR46] Cortes-Ciriano I (2023). Author correction: comprehensive analysis of chromothripsis in 2,658 human cancers using whole-genome sequencing. Nat Genet.

[CR47] Crisafulli G, Siravegna G (2023). Editorial: the impact of genetics on CRC therapy: from adaptive mutability to drug resistance. Front Oncol.

[CR48] Cuadrado M (2006). ATM regulates ATR chromatin loading in response to DNA double-strand breaks. J Exp Med.

[CR49] Cucinotta FA (2022). Flying without a net: space radiation cancer risk predictions without a gamma-ray basis. Int J Mol Sci.

[CR50] Cucinotta FA (2020). Benchmarking risk predictions and uncertainties in the NSCR model of GCR cancer risks with revised low let risk coefficients. Life Sci Space Res (amst).

[CR51] Cunniffe S (2014). Increased mutability and decreased repairability of a three-lesion clustered DNA-damaged site comprised of an AP site and bi-stranded 8-oxoG lesions. Int J Radiat Biol.

[CR52] Cunniffe S (2014). Reduced repair capacity of a DNA clustered damage site comprised of 8-oxo-7,8-dihydro-2'-deoxyguanosine and 2-deoxyribonolactone results in an increased mutagenic potential of these lesions. Mutat Res.

[CR53] Dagogo-Jack I, Shaw AT (2018). Tumour heterogeneity and resistance to cancer therapies. Nat Rev Clin Oncol.

[CR54] Dai C (2018). The heat-shock, or HSF1-mediated proteotoxic stress, response in cancer: from proteomic stability to oncogenesis. Philos Trans R Soc Lond B Biol Sci.

[CR55] Danforth JM, Provencher L, Goodarzi AA (2022). Chromatin and the cellular response to particle radiation-induced oxidative and clustered DNA damage. Front Cell Dev Biol.

[CR56] Darafsheh A (2021). spread-out Bragg peak proton FLASH irradiation using a clinical synchrocyclotron: PROOF of concept and ion chamber characterization. Med Phys.

[CR57] Datta K, Neumann RD, Winters TA (2005). Characterization of complex apurinic/apyrimidinic-site clustering associated with an authentic site-specific radiation-induced DNA double-strand break. Proc Natl Acad Sci U S A.

[CR58] Datta K (2013). Heavy ion radiation exposure triggered higher intestinal tumor frequency and greater beta-catenin activation than gamma radiation in APC(Min/+) mice. PLoS ONE.

[CR59] de Kruijff RM, Wolterbeek HT, Denkova AG (2015). A critical review of alpha radionuclide therapy-how to deal with recoiling daughters?. Pharmaceuticals (basel).

[CR60] Debela DT (2021). New approaches and procedures for cancer treatment: Current perspectives. SAGE Open Med.

[CR61] Deng S (2022). Targeting the DNA damage response and DNA repair pathways to enhance radiosensitivity in colorectal cancer. Cancers (basel).

[CR62] Deorukhkar A, Krishnan S (2010). Targeting inflammatory pathways for tumor radiosensitization. Biochem Pharmacol.

[CR63] Dietlein F, Reinhardt HC (2014). Molecular pathways: exploiting tumor-specific molecular defects in DNA repair pathways for precision cancer therapy. Clin Cancer Res.

[CR64] Dietlein F, Thelen L, Reinhardt HC (2014). Cancer-specific defects in DNA repair pathways as targets for personalized therapeutic approaches. Trends Genet.

[CR65] Dongre A, Weinberg RA (2019). New insights into the mechanisms of epithelial-mesenchymal transition and implications for cancer. Nat Rev Mol Cell Biol.

[CR66] Dracham CB, Shankar A, Madan R (2018). Radiation induced secondary malignancies: a review article. Radiat Oncol J.

[CR67] Du TQ (2022). Does particle radiation have superior radiobiological advantages for prostate cancer cells? a systematic review of in vitro studies. Eur J Med Res.

[CR68] Eccles LJ, O'Neill P, Lomax ME (2011). Delayed repair of radiation induced clustered DNA damage: friend or foe?. Mutat Res.

[CR69] Eckelmann BJ (2020). XRCC1 promotes replication restart, nascent fork degradation and mutagenic DNA repair in BRCA2-deficient cells. NAR Cancer.

[CR70] Eot-Houllier G (2005). Processing of a complex multiply damaged DNA site by human cell extracts and purified repair proteins. Nucleic Acids Res.

[CR71] Facoetti A (2019). The role of particle therapy in the risk of radio-induced second tumors: a review of the literature. Anticancer Res.

[CR72] Ferlay J (2021). Cancer statistics for the year 2020: an overview. Int J Cancer.

[CR73] Fossati P (2018). Radiobiological issues in prospective carbon ion therapy trials. Med Phys.

[CR74] Fu X (2024). Mechanism of PARP inhibitor resistance and potential overcoming strategies. Genes Dis.

[CR75] Fuchs RP (2016). Tolerance of lesions in *E coli*: chronological competition between translesion synthesis and damage avoidance. DNA Repair (amst).

[CR76] Fujisawa H (2015). VE-821, an ATR inhibitor, causes radiosensitization in human tumor cells irradiated with high LET radiation. Radiat Oncol.

[CR77] Gameiro SR (2016). Tumor cells surviving exposure to proton or photon radiation share a common immunogenic modulation signature, rendering them more sensitive to T Cell-mediated killing. Int J Radiat Oncol Biol Phys.

[CR78] Georgakilas AG (2004). Processing of bistranded abasic DNA clusters in gamma-irradiated human hematopoietic cells. Nucleic Acids Res.

[CR79] Georgakilas AG, O'Neill P, Stewart RD (2013). Induction and repair of clustered DNA lesions: what do we know so far?. Radiat Res.

[CR80] Georgoulis A (2017). Genome instability and gammaH2AX. Int J Mol Sci.

[CR81] Gerelchuluun A (2015). The major DNA repair pathway after both proton and carbon-ion radiation is NHEJ, but the HR pathway is more relevant in carbon ions. Radiat Res.

[CR82] Glanzer JG (2014). RPA inhibition increases replication stress and suppresses tumor growth. Cancer Res.

[CR83] Goetz G (2019). Health technology assessment of carbon-ion beam radiotherapy: a systematic review of clinical effectiveness and safety for 54 oncological indications in 12 tumour regions. Anticancer Res.

[CR84] Goodarzi AA, Block WD, Lees-Miller SP (2003). The role of ATM and ATR in DNA damage-induced cell cycle control. Prog Cell Cycle Res.

[CR85] Goodhead DT, Nikjoo H (1989). Track structure analysis of ultrasoft X-rays compared to high- and low-LET radiations. Int J Radiat Biol.

[CR86] Hada M, Georgakilas AG (2008). Formation of clustered DNA damage after high-LET irradiation: a review. J Radiat Res.

[CR87] Hanna C (2020). Treatment of newly diagnosed glioblastoma in the elderly: a network meta-analysis. Cochrane Database Syst Rev.

[CR88] Hanscom T, McVey M (2020). Regulation of error-prone DNA double-strand break repair and its impact on genome evolution. Cells.

[CR89] Hanssen-Bauer A (2012). X-ray repair cross complementing protein 1 in base excision repair. Int J Mol Sci.

[CR90] Harper JV, Anderson JA, O'Neill P (2010). Radiation induced DNA DSBs: Contribution from stalled replication forks?. DNA Repair (amst).

[CR91] Harrison L, Hatahet Z, Wallace SS (1999). In vitro repair of synthetic ionizing radiation-induced multiply damaged DNA sites. J Mol Biol.

[CR92] Hayashi K (2019). Feasibility of Re-irradiation using carbon ions for recurrent head and neck malignancies after carbon-ion radiotherapy. Radiother Oncol.

[CR93] Hays E (2020). The SWI/SNF ATPase BRG1 stimulates DNA end resection and homologous recombination by reducing nucleosome density at DNA double strand breaks and by promoting the recruitment of the CtIP nuclease. Cell Cycle.

[CR94] He J (2022). The roles of EXO1 and RPA1 polymorphisms in prognosis of lung cancer patients treated with platinum-based chemotherapy. Dis Markers.

[CR95] Higuchi T (2015). CTLA-4 blockade synergizes therapeutically with PARP inhibition in BRCA1-deficient ovarian cancer. Cancer Immunol Res.

[CR96] Hirai T (2012). Radiosensitization effect of poly(ADP-ribose) polymerase inhibition in cells exposed to low and high liner energy transfer radiation. Cancer Sci.

[CR97] Hsu CW (2022). A combinatorial system to examine the enzymatic repair of multiply damaged DNA substrates. Nucleic Acids Res.

[CR98] Huang RX, Zhou PK (2020). DNA damage response signaling pathways and targets for radiotherapy sensitization in cancer. Signal Transduct Target Ther.

[CR99] Huang L (2023). High-throughput sequencing reveals Jatrorrhizine inhibits colorectal cancer growth by ferroptosis-related genes. BMC Med Genomics.

[CR100] Igarashi T (2023). An ATR-PrimPol pathway confers tolerance to oncogenic KRAS-induced and heterochromatin-associated replication stress. Nat Commun.

[CR101] Jackson SP (2002). Sensing and repairing DNA double-strand breaks. Carcinogenesis.

[CR102] Jefferson T (2019). Hadrontherapy for cancer. an overview of HTA reports and ongoing studies. Recenti Prog Med.

[CR103] Jiang Y, Wang Y, Wang Y (2009). In vitro replication and repair studies of tandem lesions containing neighboring thymidine glycol and 8-oxo-7,8-dihydro-2'-deoxyguanosine. Chem Res Toxicol.

[CR104] Jokar S (2022). The footprint of exosomes in the radiation-induced bystander effects. Bioengineering (basel).

[CR105] Kabakov AE, Yakimova AO (2021). Hypoxia-induced cancer cell responses driving radioresistance of hypoxic tumors: approaches to targeting and radiosensitizing. Cancers (basel).

[CR106] Kamada T (2015). Carbon ion radiotherapy in Japan: an assessment of 20 years of clinical experience. Lancet Oncol.

[CR107] Kamaletdinova T, Fanaei-Kahrani Z, Wang ZQ (2019). The enigmatic function of PARP1: from PARYLATION activity to PAR readers. Cells.

[CR108] Karwowski BT (2019). The influence of (5'R)- and (5'S)-5',8-Cyclo-2'-deoxyadenosine on UDG and hAPE1 activity. tandem lesions are the base excision repair system's nightmare. Cells.

[CR109] Karwowski BT (2021). (5'S) 5',8-Cyclo-2'-deoxyadenosine cannot Stop BER. clustered DNA lesion studies. Int J Mol Sci.

[CR110] Kazmierczak-Baranska J (2021). Effects of 5',8'-Cyclo-2'-deoxypurines on the base excision repair of clustered DNA lesions in nuclear extracts of the XPC cell line. Cells.

[CR111] Kennedy EM (2018). Galactic cosmic radiation induces persistent epigenome alterations relevant to human lung cancer. Sci Rep.

[CR112] Khan T (2020). Autophagy modulators for the treatment of oral and esophageal squamous cell carcinomas. Med Res Rev.

[CR113] Kiseleva V (2022). Particle therapy: clinical applications and biological effects. Life (basel).

[CR114] Kong X (2021). Relationship between p53 status and the bioeffect of ionizing radiation. Oncol Lett.

[CR115] Kozmin SG (2021). Dissecting highly mutagenic processing of complex clustered DNA damage in yeast *Saccharomyces cerevisiae*. Cells.

[CR116] Kraus KM, Combs SE (2019). The risk of subsequent primary cancer after radiotherapy of localised prostate cancer with carbon ions may be reduced compared to treatment with photons. Strahlenther Onkol.

[CR117] Kudryavtsev VA (2017). Induction of Hsp70 in tumor cells treated with inhibitors of the Hsp90 activity: a predictive marker and promising target for radiosensitization. PLoS ONE.

[CR118] Kumar K (2023). High-LET-radiation-induced persistent DNA damage response signaling and gastrointestinal cancer development. Curr Oncol.

[CR119] Lee Y (2016). The purine scaffold Hsp90 inhibitor PU-H71 sensitizes cancer cells to heavy ion radiation by inhibiting DNA repair by homologous recombination and non-homologous end joining. Radiother Oncol.

[CR120] Li F, Sethi G (2010). Targeting transcription factor NF-kappaB to overcome chemoresistance and radioresistance in cancer therapy. Biochim Biophys Acta.

[CR121] Li HK (2016). PU-H71, a novel Hsp90 inhibitor, as a potential cancer-specific sensitizer to carbon-ion beam therapy. J Radiat Res.

[CR122] Li S (2019). Genetic variants in RPA1 associated with the response to oxaliplatin-based chemotherapy in colorectal cancer. J Gastroenterol.

[CR123] Li F (2021). Shift in G(1)-checkpoint from ATM-alone to a cooperative ATM Plus ATR regulation with increasing dose of radiation. Cells.

[CR124] Li Z (2021). Characterization of exosome release and extracellular vesicle-associated miRNAs for human bronchial epithelial cells irradiated with high charge and energy ions. Life Sci Space Res (amst).

[CR125] Li M (2021). Clinical efficacy and safety of proton and carbon ion radiotherapy for prostate cancer: a systematic review and meta-analysis. Front Oncol.

[CR126] Lin YF (2018). The relative biological effect of spread-out bragg peak protons in sensitive and resistant tumor cells. Int J Part Ther.

[CR127] Liu X (2018). Genistein sensitizes glioblastoma cells to carbon ions via inhibiting DNA-PKcs phosphorylation and subsequently repressing NHEJ and delaying HR repair pathways. Radiother Oncol.

[CR128] Liu YP (2021). Molecular mechanisms of chemo- and radiotherapy resistance and the potential implications for cancer treatment. MedComm.

[CR129] Liu J (2022). Wnt/beta-catenin signalling: function, biological mechanisms, and therapeutic opportunities. Signal Transduct Target Ther.

[CR130] Lobrich M, Jeggo PA (2005). Harmonising the response to DSBs: a new string in the ATM bow. DNA Repair (amst).

[CR131] Lomax ME, Cunniffe S, O'Neill P (2004). 8-OxoG retards the activity of the ligase III/XRCC1 complex during the repair of a single-strand break, when present within a clustered DNA damage site. DNA Repair (amst).

[CR132] Lomax ME, Cunniffe S, O'Neill P (2004). Efficiency of repair of an abasic site within DNA clustered damage sites by mammalian cell nuclear extracts. Biochemistry.

[CR133] Lorat Y (2015). Nanoscale analysis of clustered DNA damage after high-LET irradiation by quantitative electron microscopy–the heavy burden to repair. DNA Repair (amst).

[CR134] Lorat Y (2016). Clustered double-strand breaks in heterochromatin perturb DNA repair after high linear energy transfer irradiation. Radiother Oncol.

[CR135] Luitel K (2018). Proton radiation-induced cancer progression. Life Sci Space Res (amst).

[CR136] Ma H (2015). Combining carbon ion irradiation and non-homologous end-joining repair inhibitor NU7026 efficiently kills cancer cells. Radiat Oncol.

[CR137] Malouff TD (2020). Carbon ion therapy: a modern review of an emerging technology. Front Oncol.

[CR138] Malyarchuk S, Harrison L (2005). DNA repair of clustered uracils in HeLa cells. J Mol Biol.

[CR139] Malyarchuk S (2003). The mutation frequency of 8-oxo-7,8-dihydroguanine (8-oxodG) situated in a multiply damaged site: comparison of a single and two closely opposed 8-oxodG in Escherichia coli. DNA Repair (amst).

[CR140] Malyarchuk S (2004). Two clustered 8-oxo-7,8-dihydroguanine (8-oxodG) lesions increase the point mutation frequency of 8-oxodG, but do not result in double strand breaks or deletions in Escherichia coli. Nucleic Acids Res.

[CR141] Malyarchuk S, Castore R, Harrison L (2008). DNA repair of clustered lesions in mammalian cells: involvement of non-homologous end-joining. Nucleic Acids Res.

[CR142] Malyarchuk S, Castore R, Harrison L (2009). Apex1 can cleave complex clustered DNA lesions in cells. DNA Repair (amst).

[CR143] Mardanshahi A (2021). The PI3K/AKT/mTOR signaling pathway inhibitors enhance radiosensitivity in cancer cell lines. Mol Biol Rep.

[CR144] Marechal A, Zou L (2013). DNA damage sensing by the ATM and ATR kinases. Cold Spring Harb Perspect Biol.

[CR145] Marini V (2023). MUS81 cleaves TOP1-derived lesions and other DNA-protein cross-links. BMC Biol.

[CR146] Masumura K (2002). Heavy-ion-induced mutations in the gpt delta transgenic mouse: comparison of mutation spectra induced by heavy-ion, X-ray, and gamma-ray radiation. Environ Mol Mutagen.

[CR147] McKenzie GJ (2000). The SOS response regulates adaptive mutation. Proc Natl Acad Sci U S A.

[CR148] Michalettou TD (2021). A Meta-analysis of the effects of high-LET ionizing radiations in human gene expression. Life (basel).

[CR149] Mishra MV (2017). Establishing evidence-based indications for proton therapy: an overview of current clinical trials. Int J Radiat Oncol Biol Phys.

[CR150] Miyasaka A (2015). PI3K/mTOR pathway inhibition overcomes radioresistance via suppression of the HIF1-alpha/VEGF pathway in endometrial cancer. Gynecol Oncol.

[CR151] Mladenov E (2019). Radiation-dose-dependent functional synergisms between ATM, ATR and DNA-PKcs in checkpoint control and resection in G(2)-phase. Sci Rep.

[CR152] Mladenov E (2019). DNA-PKcs and ATM epistatically suppress DNA end resection and hyperactivation of ATR-dependent G(2)-checkpoint in S-phase irradiated cells. Sci Rep.

[CR153] Mladenova V (2022). DNA damage clustering after ionizing radiation and consequences in the processing of chromatin breaks. Molecules.

[CR154] Mladenova V (2022). The high toxicity of DSB-clusters modelling high-LET-DNA damage derives from inhibition of c-NHEJ and promotion of alt-EJ and SSA despite increases in HR. Front Cell Dev Biol.

[CR155] Mohamad O (2017). Carbon ion radiotherapy: a review of clinical experiences and preclinical research, with an emphasis on DNA damage/repair. Cancers (basel).

[CR156] Mohamad O (2019). Risk of subsequent primary cancers after carbon ion radiotherapy, photon radiotherapy, or surgery for localised prostate cancer: a propensity score-weighted, retrospective, cohort study. Lancet Oncol.

[CR157] Mohammadian Gol T, Rodemann HP, Dittmann K (2019). Depletion of Akt1 and Akt2 impairs the repair of radiation-induced DNA double strand breaks via homologous recombination. Int J Mol Sci.

[CR158] Mondini M (2020). Radiotherapy-immunotherapy combinations - perspectives and challenges. Mol Oncol.

[CR159] Moo TA (2018). Overview of breast cancer therapy. PET Clin.

[CR160] Morales-Ramirez P (2022). Induction and assessment of persistent radioresistance in murine leukocytes in vivo. Biochem Biophys Rep.

[CR161] Mourgues S, Lomax ME, O'Neill P (2007). Base excision repair processing of abasic site/single-strand break lesions within clustered damage sites associated with XRCC1 deficiency. Nucleic Acids Res.

[CR162] Mozaffari NL, Pagliarulo F, Sartori AA (2021). Human CtIP: A 'double agent' in DNA repair and tumorigenesis. Semin Cell Dev Biol.

[CR163] Musha A (2022). Carbon-ion radiotherapy for inoperable head and neck bone and soft-tissue sarcoma: prospective observational study. Anticancer Res.

[CR164] Nakashima H, Takatsu T, Imai R (2022). Radiation-induced osteosarcoma in the pubic bone after proton radiotherapy for prostate cancer: a case report. J Rural Med.

[CR165] Naldiga S (2020). Mutagenic effects of a 2-deoxyribonolactone-thymine glycol Tandem DNA lesion in human cells. Biochemistry.

[CR166] Nayak S (2020). Inhibition of the translesion synthesis polymerase REV1 exploits replication gaps as a cancer vulnerability. Sci Adv.

[CR167] Nayak S, Calvo JA, Cantor SB (2021). Targeting translesion synthesis (TLS) to expose replication gaps, a unique cancer vulnerability. Expert Opin Ther Targets.

[CR168] Nickoloff JA, Sharma N, Taylor L (2020). Clustered DNA double-strand breaks: biological effects and relevance to cancer radiotherapy. Genes (basel).

[CR169] Nickoloff JA (2022). Nucleases and Co-factors in DNA replication stress responses. DNA (basel).

[CR170] Nikitaki Z (2016). Measurement of complex DNA damage induction and repair in human cellular systems after exposure to ionizing radiations of varying linear energy transfer (LET). Free Radic Res.

[CR171] Nikjoo H (1998). Track structure in radiation biology: theory and applications. Int J Radiat Biol.

[CR172] Niu Y (2013). XRCC1 deficiency increased the DNA damage induced by gamma-ray in HepG2 cell: involvement of DSB repair and cell cycle arrest. Environ Toxicol Pharmacol.

[CR173] Noordermeer SM (2018). The shieldin complex mediates 53BP1-dependent DNA repair. Nature.

[CR174] Nusawardhana A (2024). USP1-dependent nucleolytic expansion of PRIMPOL-generated nascent DNA strand discontinuities during replication stress. Nucleic Acids Res.

[CR175] Oden J, Eriksson K, Toma-Dasu I (2017). Inclusion of a variable RBE into proton and photon plan comparison for various fractionation schedules in prostate radiation therapy. Med Phys.

[CR176] Okada T (2010). Carbon ion radiotherapy: clinical experiences at National Institute of Radiological Science (NIRS). J Radiat Res.

[CR177] Okayasu R (2012). Repair of DNA damage induced by accelerated heavy ions–a mini review. Int J Cancer.

[CR178] Okayasu R (2006). Repair of DNA damage induced by accelerated heavy ions in mammalian cells proficient and deficient in the non-homologous end-joining pathway. Radiat Res.

[CR179] Paap B, Wilson DM, Sutherland BM (2008). Human abasic endonuclease action on multilesion abasic clusters: implications for radiation-induced biological damage. Nucleic Acids Res.

[CR180] Paganetti H (2022). Mechanisms and review of clinical evidence of variations in relative biological effectiveness in proton therapy. Int J Radiat Oncol Biol Phys.

[CR181] Paganetti H (2002). Relative biological effectiveness (RBE) values for proton beam therapy. Int J Radiat Oncol Biol Phys.

[CR182] Pai JT (2021). Induction of G2/M Cell cycle arrest via p38/p21(Waf1/Cip1)-dependent signaling pathway activation by bavachinin in non-small-cell lung cancer cells. Molecules.

[CR183] Park HA (2022). Validation of Genetic Markers Associated with Survival in Colorectal Cancer Patients Treated with Oxaliplatin-Based Chemotherapy. Cancer Epidemiol Biomarkers Prev.

[CR184] Patel ZS (2020). Red risks for a journey to the red planet: the highest priority human health risks for a mission to Mars. NPJ Microgravity.

[CR185] Peitzsch C (2019). Cancer stem cells in radiation response: current views and future perspectives in radiation oncology. Int J Radiat Biol.

[CR186] Peng M (2018). Opposing roles of FANCJ and HLTF protect forks and restrain replication during stress. Cell Rep.

[CR187] Perdyan A (2024). Chromosomal positioning and epigenetic architecture influence DNA methylation patterns triggered by galactic cosmic radiation. Sci Rep.

[CR188] Pucci C, Martinelli C, Ciofani G (2019). Innovative approaches for cancer treatment: current perspectives and new challenges. Ecancermedicalscience.

[CR189] Ramaekers BL (2011). Systematic review and meta-analysis of radiotherapy in various head and neck cancers: comparing photons, carbon-ions and protons. Cancer Treat Rev.

[CR190] Ray S (2018). Comparing photon and charged particle therapy using DNA damage biomarkers. Int J Part Ther.

[CR191] Roobol SJ (2020). Comparison of high- and low-LET radiation-induced DNA double-strand break processing in living cells. Int J Mol Sci.

[CR192] Rose M (2020). PARP inhibitors: clinical relevance, mechanisms of action and tumor resistance. Front Cell Dev Biol.

[CR193] Ruff SE (2020). Roles for MDC1 in cancer development and treatment. DNA Repair (amst).

[CR194] Russo M (2019). Adaptive mutability of colorectal cancers in response to targeted therapies. Science.

[CR195] Rycaj K, Tang DG (2014). Cancer stem cells and radioresistance. Int J Radiat Biol.

[CR196] Sadeghi Moghadam M (2023). Chromosomal instability in various generations of human mesenchymal stem cells following the therapeutic radiation. Stem Cells Int.

[CR197] Sage E, Harrison L (2011). Clustered DNA lesion repair in eukaryotes: relevance to mutagenesis and cell survival. Mutat Res.

[CR198] Sage E, Shikazono N (2017). Radiation-induced clustered DNA lesions: repair and mutagenesis. Free Radic Biol Med.

[CR199] Sai S, Koto M, Yamada S (2023). Basic and translational research on carbon-ion radiobiology. Am J Cancer Res.

[CR200] Saldanha J (2023). The adaptive mechanisms and checkpoint responses to a stressed DNA replication fork. Int J Mol Sci.

[CR201] Sallmyr A, Tomkinson AE (2018). Repair of DNA double-strand breaks by mammalian alternative end-joining pathways. J Biol Chem.

[CR202] Sarbajna S, Davies D, West SC (2014). Roles of SLX1-SLX4, MUS81-EME1, and GEN1 in avoiding genome instability and mitotic catastrophe. Genes Dev.

[CR203] Sato K, Shimokawa T, Imai T (2019). Difference in acquired radioresistance induction between repeated photon and particle irradiation. Front Oncol.

[CR204] Schilling D (2015). Sensitizing tumor cells to radiation by targeting the heat shock response. Cancer Lett.

[CR205] Scully R (2019). DNA double-strand break repair-pathway choice in somatic mammalian cells. Nat Rev Mol Cell Biol.

[CR206] Seyedin SN (2020). Combination therapy with radiation and PARP inhibition enhances responsiveness to Anti-PD-1 therapy in colorectal tumor models. Int J Radiat Oncol Biol Phys.

[CR207] Shiba S (2022). Safety and efficacy of re-irradiation with carbon-ion radiotherapy for pelvic recurrence of rectal cancer after preoperative chemoradiotherapy: a retrospective analysis. In Vivo.

[CR208] Shiba S (2022). Long-term survival of two patients with inoperable post-irradiation osteosarcoma treated with carbon-ion radiotherapy: a case report. Radiat Oncol.

[CR209] Shiba S (2023). Carbon-ion radiotherapy for inoperable solitary fibrous tumor of the skull base: a case report. In Vivo.

[CR210] Shikazono N, Akamatsu K (2020). Strand with mutagenic lesion is preferentially used as a template in the region of a bi-stranded clustered DNA damage site in *Escherichia coli*. Sci Rep.

[CR211] Shikazono N (2006). The roles of specific glycosylases in determining the mutagenic consequences of clustered DNA base damage. Nucleic Acids Res.

[CR212] Shimura T (2017). Targeting the AKT/cyclin D1 pathway to overcome intrinsic and acquired radioresistance of tumors for effective radiotherapy. Int J Radiat Biol.

[CR213] Shimura T (2011). Targeting the AKT/GSK3beta/cyclin D1/Cdk4 survival signaling pathway for eradication of tumor radioresistance acquired by fractionated radiotherapy. Int J Radiat Oncol Biol Phys.

[CR214] Shimura T (2012). Activation of the AKT/cyclin D1/Cdk4 survival signaling pathway in radioresistant cancer stem cells. Oncogenesis.

[CR215] Singh V, Das P (2013). Condensation of DNA–a putative obstruction for repair process in abasic clustered DNA damage. DNA Repair (amst).

[CR216] Smith J (2010). The ATM-Chk2 and ATR-Chk1 pathways in DNA damage signaling and cancer. Adv Cancer Res.

[CR217] Song B, Yang P, Zhang S (2024). Cell fate regulation governed by p53: Friends or reversible foes in cancer therapy. Cancer Commun (lond).

[CR218] Srivastava P, Sarma A, Chaturvedi CM (2018). Targeting DNA repair with PNKP inhibition sensitizes radioresistant prostate cancer cells to high LET radiation. PLoS ONE.

[CR219] Stover EH (2016). Biomarkers of response and resistance to DNA repair targeted therapies. Clin Cancer Res.

[CR220] Strigari L (2018). Tumour control in ion beam radiotherapy with different ions in the presence of hypoxia: an oxygen enhancement ratio model based on the microdosimetric kinetic model. Phys Med Biol.

[CR221] Suman S (2013). Therapeutic and space radiation exposure of mouse brain causes impaired DNA repair response and premature senescence by chronic oxidant production. Aging (albany NY).

[CR222] Suman S (2018). The effect of carbon irradiation is associated with greater oxidative stress in mouse intestine and colon relative to gamma-rays. Free Radic Res.

[CR223] Sung H (2021). Global cancer statistics 2020: GLOBOCAN estimates of incidence and mortality worldwide for 36 cancers in 185 countries. CA Cancer J Clin.

[CR224] Tang N (2019). Influence of chromatin compaction on simulated early radiation-induced DNA damage using Geant4-DNA. Med Phys.

[CR225] Tian H (2015). DNA damage response–a double-edged sword in cancer prevention and cancer therapy. Cancer Lett.

[CR226] Timm S (2018). Clustered DNA damage concentrated in particle trajectories causes persistent large-scale rearrangements in chromatin architecture. Radiother Oncol.

[CR227] Tinganelli W, Durante M (2020). Carbon ion radiobiology. Cancers (basel).

[CR228] Tomimatsu N (2017). DNA-damage-induced degradation of EXO1 exonuclease limits DNA end resection to ensure accurate DNA repair. J Biol Chem.

[CR229] Tomizawa K (2023). Repeated carbon-ion radiation therapy for intrahepatic recurrent hepatocellular carcinoma. Int J Radiat Oncol Biol Phys.

[CR230] Tomsia M (2024). Long-term space missions' effects on the human organism: what we do know and what requires further research. Front Physiol.

[CR231] Tonzi P, Huang TT (2019). Role of Y-family translesion DNA polymerases in replication stress: Implications for new cancer therapeutic targets. DNA Repair (amst).

[CR232] Trego KS (2016). Non-catalytic roles for XPG with BRCA1 and BRCA2 in homologous recombination and genome stability. Mol Cell.

[CR233] Tsujii H (2004). Overview of clinical experiences on carbon ion radiotherapy at NIRS. Radiother Oncol.

[CR234] Vaitsiankova A (2022). PARP inhibition impedes the maturation of nascent DNA strands during DNA replication. Nat Struct Mol Biol.

[CR235] Venkadakrishnan J (2023). Implications of translesion DNA synthesis polymerases on genomic stability and human health. Mol Cell Biol.

[CR236] Venkhataraman R (2001). Enzymatic processing of DNA containing tandem dihydrouracil by endonucleases III and VIII. Nucleic Acids Res.

[CR237] Vertemara J, Tisi R (2023). Dynamic properties of the DNA damage response Mre11/Rad50 complex. Int J Mol Sci.

[CR238] Visnes T (2018). Targeting BER enzymes in cancer therapy. DNA Repair (amst).

[CR239] Voronina N (2020). The landscape of chromothripsis across adult cancer types. Nat Commun.

[CR240] Wang H, Xu X (2017). Microhomology-mediated end joining: new players join the team. Cell Biosci.

[CR241] Wang H (2010). Characteristics of DNA-binding proteins determine the biological sensitivity to high-linear energy transfer radiation. Nucleic Acids Res.

[CR242] Wang H (2014). Distinct roles of Ape1 protein, an enzyme involved in DNA repair, in high or low linear energy transfer ionizing radiation-induced cell killing. J Biol Chem.

[CR243] Wang JS, Wang HJ, Qian HL (2018). Biological effects of radiation on cancer cells. Mil Med Res.

[CR244] Watanabe R, Rahmanian S, Nikjoo H (2015). Spectrum of radiation-induced clustered non-DSB damage - a Monte Carlo track structure modeling and calculations. Radiat Res.

[CR245] Weber AM, Ryan AJ (2015). ATM and ATR as therapeutic targets in cancer. Pharmacol Ther.

[CR246] Whalen MK (2008). Specific ATM-mediated phosphorylation dependent on radiation quality. Radiat Res.

[CR247] Wilkinson B, Hill MA, Parsons JL (2023). The cellular response to complex DNA damage induced by ionising radiation. Int J Mol Sci.

[CR248] Wilson SH, Kunkel TA (2000). Passing the baton in base excision repair. Nat Struct Biol.

[CR249] Wu Y (2015). EEPD1 rescues stressed replication forks and maintains genome stability by promoting end resection and homologous recombination repair. PLoS Genet.

[CR250] Wu Y (2023). Molecular mechanisms of tumor resistance to radiotherapy. Mol Cancer.

[CR251] Wu W (2023). Mitotic DNA synthesis in response to replication stress requires the sequential action of DNA polymerases zeta and delta in human cells. Nat Commun.

[CR252] Wyatt HD (2013). Coordinated actions of SLX1-SLX4 and MUS81-EME1 for Holliday junction resolution in human cells. Mol Cell.

[CR253] Xie M (2020). Bcl2-induced DNA replication stress promotes lung carcinogenesis in response to space radiation. Carcinogenesis.

[CR254] Xu X (2021). Structure specific DNA recognition by the SLX1-SLX4 endonuclease complex. Nucleic Acids Res.

[CR255] Xue L (2009). Regulation of ATM in DNA double strand break repair accounts for the radiosensitivity in human cells exposed to high linear energy transfer ionizing radiation. Mutat Res.

[CR256] Yajima H (2013). The complexity of DNA double strand breaks is a critical factor enhancing end-resection. DNA Repair (amst).

[CR257] Yang J (2018). Salvage carbon ion radiation therapy for locally recurrent or radiation-induced second primary sarcoma of the head and neck. J Cancer.

[CR258] Yang F, Hu Z, Guo Z (2022). Small-molecule inhibitors targeting FEN1 for cancer therapy. Biomolecules.

[CR259] Yang J (2022). Carbon-ion radiotherapy in the treatment of radiation-induced second primary malignancies. Ann Transl Med.

[CR260] Yatagai F (2002). Heavy-ion-induced mutations in the gpt delta transgenic mouse: effect of p53 gene knockout. Environ Mol Mutagen.

[CR261] Yeo CD (2017). The role of hypoxia on the acquisition of epithelial-mesenchymal transition and cancer stemness: a possible link to epigenetic regulation. Korean J Intern Med.

[CR262] Yock TI, Caruso PA (2012). Risk of second cancers after photon and proton radiotherapy: a review of the data. Health Phys.

[CR263] Yu F (2021). Wnt/beta-catenin signaling in cancers and targeted therapies. Signal Transduct Target Ther.

[CR264] Zellweger R (2015). Rad51-mediated replication fork reversal is a global response to genotoxic treatments in human cells. J Cell Biol.

[CR265] Zhang H (2015). HIF-1 regulates CD47 expression in breast cancer cells to promote evasion of phagocytosis and maintenance of cancer stem cells. Proc Natl Acad Sci U S A.

[CR266] Zhang J (2022). Flap endonuclease 1 and DNA-PKcs synergistically participate in stabilizing replication fork to encounter replication stress in glioma cells. J Exp Clin Cancer Res.

[CR267] Zhang Z (2022). Radiotherapy combined with immunotherapy: the dawn of cancer treatment. Signal Transduct Target Ther.

[CR268] Zhou Y, Paull TT (2013). DNA-dependent protein kinase regulates DNA end resection in concert with Mre11-Rad50-Nbs1 (MRN) and ataxia telangiectasia-mutated (ATM). J Biol Chem.

[CR269] Zhu Y (2009). Targeting DNA repair pathways: a novel approach to reduce cancer therapeutic resistance. Cancer Treat Rev.

[CR270] Zou Y (2006). Functions of human replication protein A (RPA): from DNA replication to DNA damage and stress responses. J Cell Physiol.

